# Analysis on the Relevance of Asthma Susceptibility with the Alteration of Integrin β 4 Expression

**DOI:** 10.1371/journal.pone.0095533

**Published:** 2014-04-16

**Authors:** Yang Xiang, Xiao-Yan Zhou, Yu-Rong Tan, Mei-Ling Tan, Hui-Jun Liu, Chi Liu, Xiang-Ping Qu, Xiao-Qun Qin

**Affiliations:** Department of Physiology, Xiangya School of Medicine, Central South University, Changsha, Hunan, PR China; Aix-Marseille University, France

## Abstract

Accumulated research has suggested the importance of the adhesion molecules modulation as therapeutic approach for bronchial asthma. Adhesion molecules expression alteration contributes to the pathogenesis of asthma. In order to probe the roles of expression imbalance of adhesion molecules in asthma pathogenesis, expression profiling of adhesion molecules was performed using cDNA microarray assay. The results showed that the expression pattern of adhesion molecules was altered in peripheral blood leucocytes of asthma patients. In this study, we focused on one of the abnormally expressed molecule, integrin β4, which was down-regulated in all asthma patients, to analyze the relevance of asthma susceptibility with the alteration of integrin β4 expressions. Real time PCR was used to verify the down-regulation of integrin β4 in additional 38 asthma patients. Next, the 5′flanking region of integrin β4 DNA were amplified, sequenced and site-directed mutagenesis technology in correspondent variation sites were carried out. Among 4 variation sites found in 5′ flanking region of integrin β4, 3 were related to asthma susceptibility: -nt1029 G/A, -nt 1051 G/A, and -nt 1164 G/C. A reduction of human integrin β4 promoter activity was observed at mutants of these sites. This study demonstrates that various adhesion molecules in asthma patients are abnormally expressed. Mutations in 5′ flanking region result in reduced integrin β4 expression, which is related to increased risk of asthma.

## Introduction

Asthma is a chronic allergic airway disease characterized by persistent inflammation and airway hyper-responsiveness (AHR)[1]. Genetic and environmental factors likely play significant roles in its pathogenesis, by modulating the airway inflammation and remodeling processes. Asthma airway inflammation and remodeling are characterized by inflammatory cells infiltration of the bronchial mucosa and are accompanied by structural changes including epithelial desquamation, subepithelial fibrosis, mucus hyperplasia, modification of the extracellular matrix and hypertrophy/hyperplasia of airway smooth muscle cells[2]. The adhesion molecules, membrane glycoprotein that intervene in the contact between the two adjacent cells or between the cell and the extracellular matrix, are involved in all of these processes.

Several distinct cell adhesion molecule families have recently been identified and found to be important in the inflammatory response and epithelial and endothelial homeostasis [3], [4], [5], [6]. The integrin family of adhesion molecules functions in both cell-matrix and cell-cell interactions, whereas cadherins serve as important cell-cell receptors for maintenance of epithelial integrity. The leukocyte integrins, selectins, members of the immunoglobulin supergene family, and specific carbohydrates mediate adhesive interactions between leukocytes and endothelial cells[7]. Cell adhesion molecules facilitate the adhesion of the circulating leukocytes to the vascular endothelium with the posterior transendothelial migration that contribute, by doing so, to the perpetuation of the inflammatory reaction in bronchial asthma [8].

Since the adhesion molecule family has many types with diverse functions, the issue of how homeostasis is maintained with regard to cells’ constitutive adhesion and the inflammation adhesion mechanism is far from being clear. In order to probe the relationship between adhesion molecules expression and asthma pathogenesis, expression profiling of adhesion molecules was performed using cDNA microarray assay. The results showed that there were various adhesion molecules with abnormal expressions in peripheral blood of asthma patients. In this study, we focused on one of the abnormally expressed molecule, integrin β4, which was down-regulated in all asthma patients, to analyze the relevance of asthma susceptibility with the alteration of integrin β4 expressions.

Integrin β4, a laminin-5 receptor, possesses two contrasting functions: stable adhesion and pro-invasive signaling, both encoded by its distinctive and long cytoplasmic tail. Integrin β4 promotes the assembly of distinctive adhesive junctions, the hemidesmosomes[9]. Most of the previous researches concentrated on the role of integrin β4 in cancer and cancer therapy[10], [11]. Recently, accumulating data reveal that integrin β4 participates in cell differentiation, multiplication[12], adhesion, migration[13], [14], macroautophagy[15], apoptosis and signal transduction[16] in various cell types, implying the key roles of integrin β4 in the physiological function of mammalian cells. Furthermore, the cytoplasmic domain of integrin β4 is different from that of other integrin subunits in both size and structure [17], [18]. Structure from distinct cytoplasmic domain subunit has been indicated the complex signaling pathway of integrin β4 (PKC, ERK, NF-κB), indicating it has a wide range of physiological effects[19].

We found integrin β4 expression was downregulated in leucocytes from patients with asthma and we wondered whether it was due to mutations in the promoter region of the gene coding integrin β4. Therefore we collected genomic DNA samples from the patients with asthma and analyzed the promoter regions of integrin β4 to further investigate the mechanism of its abnormal expression.

## Materials and Methods

### Ethics Statement

The study was approved by the Ethics Committee Institute of Central South University (Permit Number: CTXY-070007) and written informed consent was obtained from every adult participant. For the children participants, written informed consent was obtained from their parents.

### Participants

102 unrelated patients with asthma and 38 healthy people without a history of allergy or asthma were enrolled in the present study with a median age of 46 yrs (Table 1). All the subjects were of Han ethnicity. Patients were recruited from Xiangya hospital, Hunan pediatric hospital, and Xiangtan central hospital, Hunan, China. Asthma was diagnosed according to the criteria of the Chinese guidelines for the management of bronchial asthma (by Chinese Society of Respiratory Diseases, 2008) [20].

**Table 1:** Group characteristics.

	Healthy controls	Asthma patients
Number of individuals	38	102
Mean age (years)	37.9(13∼65)	48.6 (10∼74)
Gender ratio (male/female)	1.12	1.53
Mean FEV1 (%)		69.19 (65.9∼74.3)

Forced expiratory volume in one second (FEV1) is expressed in (%), which is defined as FEV1% of the patient divided by the average FEV1% in the population for any person of similar age, sex and body composition. Normal value is approximately 86%.

### Isolation of Peripheral Blood Leukocytes

Human fresh blood was drawn into heparin-coated vacuum tubes, and then diluted 1∶1 with 0.5 N Hanks’ buffered salt solution (HBSS). After centrifugation at 100×g for 10 min, the supernatant containing the leukocytes was collected and layered on Lymphoprep (Nycomed Pharma, Oslo, Norway). Following subsequent centrifugation at 800×g for 25 min, the leukocyte layer at the interface was collected and washed three times with HBSS.

### Microarray Studies and Analysis

RNA was extracted from leucocytes in peripheral blood of 4 normal adults and 6 asthma patients by using TRIzol Reagent. Microarray expression studies were performed using the GEArray Q Series Human Extracellular Matrix & Adhesion Molecules Gene Array (SABiosciences Corporation, USA). This microarray profiles the expression of 96 genes key to the functions of cell adhesion (showed in Table S1). A negative control (PUC18DNA and blank), and the housekeeping genes including β-actin, GAPDH, Cyclophilin A and ribose body protein L13a were spread on each chip. Examinations of the expression spectrum were accomplished in collaboration with the Shanghai Kangcheng Biological Technology Co., Lit. The expression spectrum of 4 normal adults from the control and 6 asthma patients was examined respectively. The results were scanned by scanners and transformed into pictures in a gradation TIFF format. Then the lattice of the gradation TIFF pictures was transformed into numeral data by using a ScanAlyze software package. Using the chip-supporting GEArray Analyzer software package, the background value was subtracted from the primary data and subsequent adjustment was made by using housekeeping genes. The data discussed in this publication have been deposited in NCBI’s Gene Expression Omnibus and are accessible through GEO Series accession number GSE54605 (http://www.ncbi.nlm.nih.gov/geo/query/acc.cgi?acc=GSE54605).

### Real Time PCR Measurement

RNA was extracted from peripheral blood leukocytes of additional 34 healthy person and 38 asthma patients. Reverse transcription was performed by *AMV* reverse transcriptase (QIAGEN, Gemany). PCR was then carried out using ShineSybr Real Time qPCR Kits (Shinegene, China). The primers were synthesized as follows: integrin β4: 5′-GCTCGCCAAGCACAAC-3′ (forward), 3′-TGGAAGGAAGAGGCTGC-5′(reverse); GAPDH: 5′-CCACTCCTCCACCTTTGAC-3′ (forward), 5′-ACCCTGTTGCTGTAGCCA-3′ (reverse). Briefly, 2 µl of the reverse-transcripts was added to a 25 µl PCR mixture for 40 cycles. Each cycle included 94°C for 5 s, 60°C for 30 s and 72°C for 30 s. Normalization of mRNA expression data for sample-to-sample variability in RNA input, RNA quality, and reverse transcription efficiency was achieved by comparing the copy numbers of target mRNAs with that of human GAPDH for each run.

### Western Blot Analysis

Control and asthma peripheral blood leukocytes were lysed in protease inhibitor cocktail solution (Roche, Indianapolis, INC, USA). Cell lysates (50 µg) were separated on 8%–10% sodium dodecyl sulfate-polyacrylamide gels (Bio-Rad, Hercules, CA, USA) and then transferred onto polyvinylidene fluoride membrane (Millipore, Billerica, MA, USA). Membranes were blocked with 5% BSA and incubated with mouse monoclonal anti-integrin beta 4 antibody (Abcam, ab29042) and Anti- integrin beta 2 antibody (Abcam, ab657) at 4°C overnight. After being washed, membranes were incubated with peroxidase-affinipure goat anti-mouse IgG (1∶5000; Jackson ImmunoResearch Laboratories,Inc, USA) for 1 h at room temperature. Antibody-antigen complexes were then detected using an ECL chemiluminescent detection system (Gene Co., Ltd., Hong Kong, China). Beta-Actin (Abcam, ab20272) was used as a loading control.

### PCR

DNA was extracted from leucocytes in peripheral blood of 34 healthy person and 96 asthma patients. 5′ flanking regions were amplified using primer as follows: 5′ region of integrin β4: 5′-GACCATCCCATTCACTCAAC-3′ (forward, −2235 nt∼−2210 nt), 5′- TGCACCCTTCAACAAGCT -3′ (reverse, − 143 nt∼−46 nt), 1 µl of DNA template was added to a 50 µl PCR mixture by using Taq DNA polymerase for 30 cycles. Denature temperature was set at 95°C, annealing temperature 57°C and extending temperature 72°C. The PCR products were sequenced by Shanghai Boya Company. The sequences of asthma patients were aligned with sequences of normal adults and Genebank respectively by using workbench software.

### Reporter Gene Construct, Site-directed Mutagenesis, and Reporter Gene Assay

The 5′ flanking region of human integrin β4 gene was PCR amplified (−2235∼−46 from ATG) and cloned into the Xho I and Hind III site of the pGL3-basic luciferase reporter vector (Firefly luciferase, Promega; Madison, WI). This reporter was designated as pGL3/integrin β4/luc in this study. Nucleotide identity and direction of the insert were verified by sequencing of both strands.

Site-directed mutagenesis was operated according to the kit instructions (TaKaRa MutanBEST Kit): A pair of 5′ end adjacency, 3′ end opposite primer was designed to import variation point. The PCR is carried out to amplify the pGL3/integrin β4/luc plasmid using PyrobestDNA high fidelity enzyme. The blunt-ended PCR-generated DNA fragment is self-ligated and used to transform Escherichia coli. Mutant clones (white clones) are selected and the presence of the mutation is confirmed by direct DNA sequencing.

Promoter activity of mutants of 5′ flanking region of human integrin β4 was assayed by Dual-luciferase assay system (Promega) as described previously[21]. Briefly, 16HBE14o^−^ cells[22] were seeded onto 24-well culture plates the day before transfection. On the day of transfection, each reporter vector (0.6 µg/well) was transfected into the cells using Lipofectamine 2000 reagent (Invitrogen) according to the manufacture’s protocol. For standardization, the phRL-TK vector (Renilla luciferase, Promega) (0.1 µg/well) was also transfected into the cells. Six hours after transfection, the medium was replaced. Twenty-four hours later, cells were harvested by Passive Lysis Buffer (Promega), and reporter gene assay was performed with Varioskan Flash multitechnology microplate reader (Thermo Scientific) using Dual-luciferase assay system. The results represent the average of three independent transfection assays normalized to Renilla reniformis activity (Firefly luciferase/Renilla luciferase).

### Statistical Analysis

The expression results between control and asthma were analyzed by the Mann-Whitney nonparametric test, gene variation results were analyzed by using chi-square test, and other numerical data were analyzed by analysis of variance. Datas were expressed as the mean±SE. Statistical difference between two groups was determined by t-test. *P*<0.05 was considered statistically significant.

## Results

### Study on the Expression Spectrum of Asthma-associated Adhesion Molecules

The chip results were scanned by scanners and analyzed by software packages. Those whose gene expressions were increased over 2 times were regarded as up-regulated genes, and those whose gene expressions were decreased over 0.5 times were regarded as down-regulated genes. The results showed that in comparison of asthma patients with the normal control group there were 3 up-regulated genes including Integrin αV, Collagenase-1 and TIMP3 and 14 down-regulated gene including adhesive molecules: Integrinα1, Integrinα6, Integrinα8, Integrinα10, integrin β4, catenin α-like 1; extracellular matrixes: COL1ALPHA1, MCH6/ALPHAPALPHAF3, Vitronectin; protease: MMP-7, MMP-17, uPA; and protease inhibitor: TIMP-1, TIMP2 in the examined 96 genes (showed in Table S1). The fold change of expression of integrin β4 was the biggest among these genes (Table 2). So we chose integrin β4 for the further study.

**Table 2:** Genes differentially expressed in peripheral blood leukocytes by microarray analysis in subjects with asthma compared with healthy control subjects.

GenBank	Symbol	Gene Name	Normal (n = 4)		Asthma (n = 6)		A vs N
			Mean	SD	Mean	SD	Mean fold change
NM_002210	ITGAV	Integrin aV	0.093700	0.060214	0.294158	0.125139	3.139364
NM_002421	MMP1	Collagenase-1	0.002498	0.000926	0.005926	0.004458	2.372812
NM_000362	TIMP3	TIMP3	0.024418	0.007758	0.060057	0.035373	2.459501
NM_001229	CASP9	MCH6/APAF3	0.101523628	0.044795307	0.042016571	0.01513775	0.413860017
NM_003798	CTNNAL1	Catenin alpha-like 1	0.149462301	0.013000094	0.026793578	0.01334572	0.179266462
NM_000201	ICAM1	ICAM-1	0.224703585	0.169752998	0.110040567	0.04430093	0.489714338
NM_181501	ITGA1	Integrin a1	0.083936913	0.030885648	0.03522235	0.01194558	0.419628842
NM_003637	ITGA10	Integrin a10	0.096937903	0.068613781	0.038395369	0.00911615	0.396082109
NM_000210	ITGA6	Integrin a6	0.117987022	0.063223591	0.018430151	0.00612577	0.1562049
XM_167711	ITGA8	Integrin a8	0.033332349	0.027166693	0.005238253	0.00312769	0.157152235
NM_000213	ITGB4	Integrin b4	0.093004077	0.026269593	0.005746634	0.00160742	0.061789053
NM_016155	MMP17	MMP-17	0.032310	0.018080	0.015070	0.009129	0.466424
NM_002423	MMP7	MMP-7	0.035165	0.022782	0.014475	0.005867	0.411642
NM_003254	TIMP1	TIMP1	0.0163375	0.004652504	0.001968667	0.00097557	0.120499872
NM_003255	TIMP2	TIMP2	0.276897855	0.107341465	0.118477336	0.01776673	0.427873794
NM_000638	VTN	Vitronectin	0.332934234	0.051878946	0.114459334	0.01868041	0.343789622
NM_002658	PLAU	uPA	0.005334282	0.004588615	0.0005667	0.00036557	0.10623745

This table shows the results of adhesion molecules expression spectrum of peripheral blood leukocytes assayed by cDNA microassay. The expression spectrum of 4 normal adults from the control and 6 asthma patients was examined respectively. Those whose gene expressions were increased over 2 times were regarded as up-regulated genes, and those whose gene expressions were decreased over 0.5 times were regarded as down-regulated genes. In comparson of asthma patients with the normal control group, there are 3 up-regulated genes and 14 down-regulated genes.

### Study on Integrin β4 Epression in Asthma Patients

**Figure 1:**
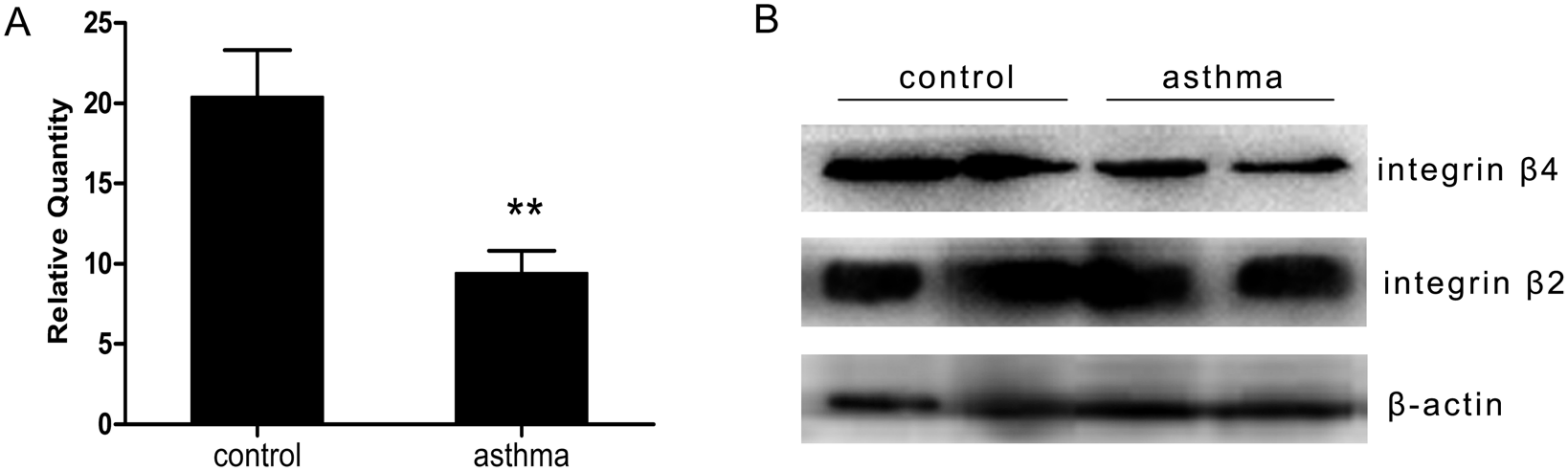
The intergrin β4 expression in peripheral blood leukocytes of asthma patients. A: Intergrin β4 mRNA expression assayed by real time PCR. Healthy control n = 34, Asthma patients n = 38. **P<0.01 versus control. B: Intergrin β4 protein expression assayed by western blot. Integrin β2 protein, which is known to be expressed at high level on leucocyte plasma membrane, was detected as a positive control. Beta-actin was used as a loading control. The lanes represent two subjects from each group.

To further verify the results of the gene array and expression of integrin β4 in asthma, real time PCR was used to test the expression level of integrin β4. The results demonstrated that the expressions of integrin β4 mRNA were remarkably down-regulated in leucocytes in peripheral blood of asthma patients compared with health control (Figure 1A ).

Western blot was done to analyze the integrin β4 protein expression in peripheral blood leukocytes. Meanwhile, the expression of integrin β2 protein, which is known to be expressed at high level on leucocyte plasma membrane, was also detected as a positive control. As shown in figure 1B , integrin β4 was down-regulated in asthma patients.

### Study on Gene Variation of Integrin β4

To probe the mechanism of integrin β4 abnormal expression, we performed direct DNA sequencing of 5′ flanking regions of integrin β4. Genomic DNA samples of leucocytes from human peripheral blood were collected from 34 healthy person and 96 asthma patients. Within these 130 DNA samples, four variation sites in 5′ flanking region of integrin β4 were found (compare with the sequence in Genebank): -nt1029 G/A (site 1), -nt 1051 G/A (site 2), -nt1151 T/G (site 3), and -nt 1164 G/C (site 4). As showed in Table 3, 35% healthy control and 85% asthma patients presented with -nt1029 G/A (site 1) variation; 66% asthma patients presented with -nt1051 G/A (site 2) variation, and 76% asthma patients presented with -nt 1164 G/C (site 4) variation, while none of the healthy control had these two variations; 38% healthy control and 43% asthma patients presented with -nt1151 T/G (site 3) variation. Frequency chi-square test showed that the site 1, 2, and 4 variations have statistical significance in asthma group.

**Table 3:** Genotype and site variation frequencies in 5′ flanking region of integrin β4 gene.

	Healthy controls	Asthma patients
	n (%)	n (%)
**Total no.**	**34**	**96**
**Site1:** G**-1029** A	12 (35%)	82 (85%)*
**Site2:** G**-1051** A	0	63 (66%)*
**Site3:** T**-1151** G	13 (38%)	41 (43%)
**Site4:** G**-1164** C	0	73 (76%)*
**Multiple site variations**		
**Site 1234**	0	23 (24%)*
**Site 124**	0	37 (39%)*
**Site 134**	0	3
**Site 12**	0	3
**Site 14**	0	5
**Site 13**	4	7
**Site 34**	0	5
**Site 1**	8	4
**Site 3**	9	3
**Site 4**	0	0
**none**	13	6

*P<0.05 versus healthy control.

Among the 96 asthma patients, 23 cases have got all the 4 sites variation; 37 cases have 3 sites variation (site 1, 2 and 4). In 34 cases of normal adult samples, no multiple sites variations were detected, but there were several cases showed single site variation (Table 3).

### The Effects of Site-directed Mutagenesis of Integrin β4 on the Expression of the Reporter Gene

For a further understanding of the contribution of the site variations in 5′ flanking region of integrin β4 to its expression, we constructed a human integrin β4 promoter-luciferase reporter, pGL3/integrin β4/luc. According to the gene variation result, three site-directed mutants were introduced into this reporter (Table 4), -nt1029 G/A (site 1), -nt 1051 G/A (site 2) and -nt 1164 G/C (site 4), to detect whether these site variations could influent mRNA expression of integrin β4. -nt1151 T/G (site 3) was not included because there was no statistical significance of this site variation in asthma group.

**Table 4:** Primers for site-directed mutation.

	Sequence of primers
Site 1 mutation F:	5′-AGAGAGAAAACAAAAAGAAAAAGA-3′
Site 1 mutation R:	5′-CTTTTTTTTTTTAAATGGAGTCTCAC-3′
	*(-1029 from ATG, G→A)*
Site 2 mutation F:	5′-GTGAGACTCCATCTAAAAAAAAAAAGAGA-3′
Site 2 mutation R:	5′-TCTGTCGCCAAGGCTGGA-3′
	*(-1051 from ATG, G→A)*
Site 4 mutation F:	5′-GGCACCTGTAATCCCAGCTACTCG-3′
Site 4 mutation R:	5′-CGCCACCACCCCCGGCTAAT-3′
	*(-1164 from ATG, G→C)*

**Figure 2:**
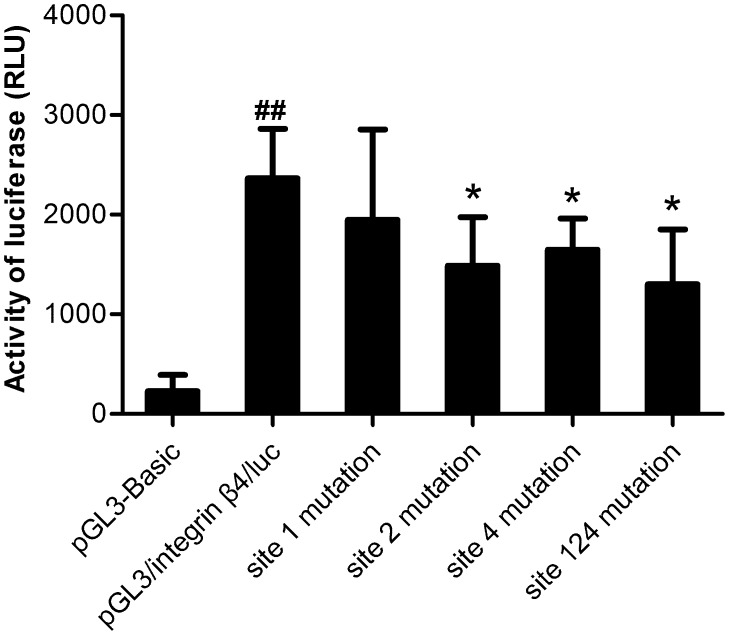
Mutants were generated in a luciferase reporter plasmid. The results showed that luciferase activities decreased after mutating the site2, site4, and remarkably decreased after mutating site124 together. Relative activities of luciferase were equal to the fluorescence intensity of firefly when activities of Renilla were equal to 1. Data are means ± SD of 4 experiments. *P<0.05 versus pGL3/integrin β4/luc, ##*P*<0.01 versus pGL3-Basic.

The above plasmids were transiently transfected into human bronchial epithelial cells (16HBE14o^−^ cell line, HBEC). Cells were collected 48 h after transfection to determine the transfection efficiency by testing luciferase chemiluminescence signal strength (RLU, relative chemiluminescence intensity). As shown in Figure 2, transient transfection with the luciferase reporter pGL3/integrin β4/luc resulted in an increase in luciferase activity relative to the empty pGL3-basic vector, demonstrating that this DNA fragment (−2235∼−46 from ATG) contains significant promoter activity in HBEC (10 fold increase).

The luciferase activities significantly decreased in the group with mutation of site 2 or site 4, and in the group with three sites mutation, site 1, 2 and 4. These results demonstrated that these variation sites in 5′ flanking region we identified in asthma patients contribute to integrin β4 promoter activity, and thus regulate integrin β4 mRNA expression.

## Discussion

Asthma is a complex disease regulated by the interplay of a large number of underlying mechanisms which contribute to the overall pathology. In recent years, several expression profiling studies have been done in peripheral blood cells of asthmatic and allergic subjects as well as lung tissue obtained from animal models or allergic airway diseases[23], [24], [25]. However, despite various breakthroughs identifying genes and gene expression patterns related to asthma, it should also be noted there has been little overlap in the novel genes identified across studies. The genetic regulation of asthma pathogenesis is still largely unknown.

In this study, we examined the expression spectrum of adhesive molecules from leukocytes in human peripheral blood by using cDNA chip technologies. The results showed that there are 3 up-regulated genes and 14 down-regulated genes in asthma patients including adhesive molecules, extracellular matrixes, protease and protease inhibitor, in comparison with the normal group. They play a critical role in mediating cell-cell, cell-tissue and cell-extracellular matrix adhesions and participate in processes of cell growth, differentiation, migration and apoptosis. The above results suggest that asthmatic patients have abnormal adhesion molecules expression profile, which has preliminarily confirmed our hypothesis that the imbalance of adhesion molecules expressions may be closely associated with asthma pathogenesis.

Among the 14 down-regulated genes, expression of integrin β4 was down-regulated in all six asthma patients, and notably, the fold change of this downregulation is the biggest. The downregulation of integrin β4 expression was verified by real-time PCR and western blot. Furthermore, our group has also demonstrated that integrin β4 was downregulated in airway epithelial cells of asthmatic patients[26].

The abnormal expressions of adhesion molecules in asthma patients are possibly caused by genetic variations. Human integrin β4 gene maps on chromosome 17q25. The organization of its exons and introns and the 5′ and 3′ flanking sequences combining 42 exons has already been elucidated recently[27], [28]. In this study, our interest in integrin β4 as a candidate gene in asthma pathogenesis is focused on the 5′ flanking region in order to explain the variation in integrin β4 levels by alterations in transcription.

Sequences analysis of 5′ flanking region of integrin β4 gene showed that there were four variation sites at -nt1029 G/A (site 1), -nt 1051 G/A (site 2), -nt1151 T/G (site 3), and -nt 1164 G/C (site 4). Statistical analysis showed that variation at sites 1, 2, and 4 were consistent with asthma susceptibility. By site-directed mutagenesis of above variation sites within pGL3/integrin β4/luc, we observed a reduction in human integrin β4 promoter activity of mutants of site 2, site 4 and site 1, 2 and 4 together.

Promoter polymorphisms may be biologically functional when vital transcription factor-binding sites are changed. In the case of integrin β4, the -nt 1164 G/C (site 4) variation lies in a putative sp1 transcription factor-binding site (analyzed by Transcription Element Search System). We noticed that site 4 variation only persent in asthmatic group, accompanied by the remarkably down-regulated expressions of integrin β4 mRNA. These results might provide some support for the speculation that -nt 1164 G/C (site 4) might be located in a region with positively transcription regulatory function. No putative transcription factor-binding site was found at site 1 and site 2. However, we discovered that mutants of site 2 reduce human integrin β4 promoter activity, indicating that complex regulation of integrin β4 expression may exist in the region.

Given that bronchial tissues are a primary site for airway inflammation and remodeling in asthmatic subjects, our group have done a series of work on the role of integrin β4 in functional homeostasis on bronchial epithelial cells. Integrin β4 is constitutively expressed in airway epithelial cells, which mediates anchorage of basal cells to ECM. The integrin β4 gene knock-out mice were shown to have cell cycle and adhesion defect[29]. As the first cell barrier to outer allergens, the airway epithelial cells showed a decreased wound repair and anti-oxidation ability after integrin β4 was downregulated[26], [30], [31]. Furthermore, downregulation of integrin β4 expression in airway epithelial cells could impair the antigen presentation ability of these cells, which further regulates airway inflammation reaction in allergic asthma[32]. All these data suggested integrin β4 is a key regulator in asthma development and a potential candidate gene for asthma treatment.

In summary, we report evidence of association of integrin β4 gene with asthma. Variation from G to C at -nt 1164, and variation from G to A at -nt 1051 in 5′ flanking region of integrin β4 gene possibly lead to the down-regulated expression of integrin β4. Further studies to elucidate the functional significance of these variations in integrin β4 gene expression would be valuable in revealing the role of this gene in asthma pathogenesis.

## Supporting Information

Table S1 GEArray Q Series Human Extracellular Matrix & Adhesion Molecules Gene Array. Microarray results of the expression of 96 genes key to the functions of cell adhesion. The expression spectrum of peripheral blood leukocytes from 4 normal adults and 6 asthma patients was examined respectively.
(XLS)
